# The Potential Roles of RNA N6-Methyladenosine in Urological Tumors

**DOI:** 10.3389/fcell.2020.579919

**Published:** 2020-09-09

**Authors:** Yang Li, Yu-zheng Ge, Luwei Xu, Zheng Xu, Quanliang Dou, Ruipeng Jia

**Affiliations:** Department of Urology, Nanjing First Hospital, Nanjing Medical University, Nanjing, China

**Keywords:** N6-methyladenosine (m^6^A), writers, erasers, readers, urological tumors

## Abstract

N6-methyladenosine (m^6^A) is regarded as the most abundant, prevalent and conserved internal mRNA modification in mammalian cells. M^6^A can be catalyzed by m^6^A methyltransferases METTL3, METTL14 and WTAP (writers), reverted by demethylases ALKBH5 and FTO (erasers), and recognized by m^6^A -binding proteins such as YTHDF1/2/3, IGF2BP1/2/3 and HNRNPA2B1 (readers). Emerging evidence suggests that m^6^A modification is significant for regulating many biological and cellular processes and participates in the pathological development of various diseases, including tumors. This article reviews recent studies on the biological function of m^6^A modification and the methylation modification of m^6^A in urological tumors.

## Introduction

In past decades, epigenetic modification has been identified to be involved in diverse biological processes and disease progression, attracting more and more attention. Epigenetics is a study of reversible, inheritable phenotypes that do not involve changes in nuclear DNA sequences (Mohammad et al., 2019), and primarily includes RNA interference, histone modification, chromatin rearrangement, DNA methylation and RNA modification (Arguello et al., 2019; McGee and Hargreaves, 2019).

RNA modification was previously regarded as occurring in high-abundance RNA species, while emerging evidence indicates that it is characterized in lowly abundant species of RNA such as non-coding RNAs and Mrna (Dominissini, 2014; Li X. et al., 2016). Among them, RNA methylation has attracted accumulating attention in recent years and N6-methyladenosine (m^6^A) is the most prevalent RNA methylation sites (Pan, 2013). M^6^A modification was firstly reported to be interrelated to the regulation of gene expression, growth and development in 1970s (Desrosiers et al., 1974; Perry et al., 1975; Chandola et al., 2015; Hsu et al., 2017), and it has been regarded as one of the most common mRNA modifications recently. Many researches have revealed that m^6^A modification mainly occured in the consensus sequence RRACH sequence (*R* = A, G; *H* = A, C, U) (Li L. J. et al., 2018), which is enriched in stop codons, 3′ untranslated region (UTR) and the last exon in non-coding RNA (Dominissini et al., 2012; Meyer et al., 2012). Besides, m^6^A is widespread in RNA of bacteria, viruses and eukaryotes (Desrosiers et al., 1974; Wang Y. et al., 2014; Deng et al., 2015; Fu et al., 2015; Greer et al., 2015; Zhang et al., 2015; Liu J. et al., 2016; Zhu et al., 2018).

M^6^A modification is reversible and catalyzed by many relevant enzymes (Batista, 2017; Dai et al., 2018). Studies have shown that m^6^A is involved in various biological and disease processes via regulating target gene expression (Chen X.Y. et al., 2019; Lan et al., 2019). M^6^A modification is associated with various diseases, such as neurological diseases (Liu E. Y et al., 2017; Salta and De Strooper, 2017) and cancers. In this review, we provide a broad overview of the relationship between RNA m^6^A methylation and urological tumors. We further highlight the possible uses in diagnostic, prognostic and therapeutic applications of m^6^A modifications for urological tumors.

## Regulators of m^6^A

**FIGURE 1:**
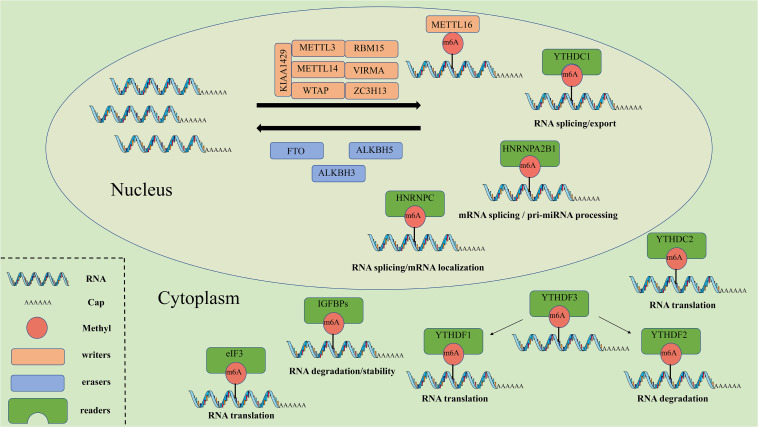
The molecular mechanism of m^6^A. M^6^A can be installed by “writers” (METTL3/14/16, WTAP, RBM15, VIRMA, KIAA1429, and ZC3H13), removed by “erasers” (FTO, ALKBH5, and ALKBH3), and recognized by “readers” (YTHDF1/2/3, YTHDC1/2, IGF2BPs, HNRNPs, and eIF3). METTL3/14/16, methyltransferase like 3/14/16; WTAP, WT1 associated protein; RBM15, RNA binding motif protein 15; VIRMA, vir like m^6^A methyltransferase associated; ZC3H13, zinc finger CCCH-type containing 13; FTO, FTO alpha-ketoglutarate dependent dioxygenase; ALKBH5, alkB homolog 5, RNA demethylase; ALKBH3, alkB homolog 3, RNA demethylase; YTHDF1/2/3, YTH N6-methyladenosine RNA binding protein 1/2/3; YTHDC1/2, YTH domain containing 1/2; IGF2BPs, insulin like growth factor 2 mRNA binding proteins; HNRNPs, heterogeneous nuclear ribonucleo proteins; eIF3, eukaryotic translation initiation factor 3 subunit.

Similar to histone modification and DNA methylation, m^6^A modification is reversible and dynamic, and influences biological functions that are primarily mediated by three types of regulators: methyltransferases (“writers”), demethylases (“erasers”) and m^6^A binding proteins (“readers”). The methyltransferase complex (MTC) can catalyze m^6^A, demethylase can remove m^6^A, while RNA reader proteins can recognize m^6^A and bind to the RNA. These proteins play an essential biological role in m^6^A modifications (Table 1, Figure 1). Cross-talk among writers, erasers and readers of m^6^A is involved in the development and progression of tumors (Deng et al., 2018; Panneerdoss et al., 2018).

**TABLE 1:** Functions of m^6^A regulators in RNA metabolism.

Type	m6A Regulators	Function	References
m^6^A writer	METTL3	Catalyzes m^6^A modification	Schwartz et al., 2014
			Zhou J. et al., 2015
–	METTL14	Forms a stable complex with METTL3	Schwartz et al., 2014
–	–	–	Zhou J. et al., 2015
	METTL16	Catalyzes m6A modification	Warda et al., 2017
–	WTAP	Contributes to the localization of METTL3-METTL14 heterodimer to the nuclear speckle	Ping et al., 2014
	RBM15	Binds the m^6^A complex and recruit it to special RNA site	Moindrot et al., 2015
–	VIRMA	Recruits the m6A complex to the special RNA site and interacts with polyadenylation	Wang T. et al., 2020
–	–	Cleavage factors CPSF5 and CPSF6	–
–	–	–	–
	ZC3H13	Bridges WTAP to the mRNA-binding factor Nito	Wen et al., 2018
m^6^A eraser	FTO	Mediates demethylation of both hm^6^A and f6A in mRNA	Basak et al., 2019
–	–	–	–
	ALKBH5	Removes m^6^A modification	Tang et al., 2018
m^6^A reader	YTHDF1	Facilitates mRNA translation efficiency	Liu J. et al., 2020
–	–	–	–
	YTHDF2	Promotes mRNA degradation	Zhou J. et al., 2015
–	YTHDF3	Enhances translation and degradation by interacting with YTHDF1 and YTHDF2	Shi et al., 2017
–	–	–	Li A. et al., 2017
	YTHDC1	Recruits the RNA splicing and controls the nuclear export	Roundtree et al., 2017b
–	YTHDC2	Interacts with RNA helicase and increases the translation efficiency of target RNA	Mao et al., 2019
–	–	–	–
	IGF2BPs	Recruits RNA stabilizers	Huang H. et al., 2018
–	HNRNPA2B1	Mediates mRNA splicing and primary microRNA processing	Alarcon et al., 2015
–	–	–	–
	HNRNPC	Influences alternative splicing and mRNA localization	Guichard et al., 2012
–	EIF3	Facilitates cap-independent translation	Meyer et al., 2015

### Methyltransferases (“Writers”)

MTC has been identified to regulate the installation of m^6^A and Methyltransferase-like 3 (METTL3), METTL14, and Wilms tumor 1-associated protein (WTAP) have been proved as the core components of this complex (Ping et al., 2014; Schwartz et al., 2014; Zhou J. et al., 2015). METTL3 is an Sadenosyl methionine (SAM)-binding protein and regarded as a major catalytic enzyme with functions reminiscent of the N6-adenine methyltransferase system (Barbieri et al., 2017). Besides, METTL3 is highly conserved in eukaryotes from yeast to humans (Bokar et al., 1997). WTAP can also increase the binding ability of METTL3, thus regulating recruitment of the complex to mRNA targets (Ping et al., 2014). METTL14 could form a stable complex with METTL3 and both of them contain a SAM-binding motif. With the help of WTAP, METTL3-METTL14 could colocalize in nuclear speckles and form a heterodimer, so as to participate in catalytic activity (Liu J. et al., 2014; Zhao X. et al., 2014). Besides, VIRMA, RBM15, ZC3H13 and KIAA1429 are the new components of the m^6^A “writer” complex (Moindrot et al., 2015; Wang X. et al., 2016; Deng et al., 2018; Wen et al., 2018).

## METTL3

The writer METTL3 has been identified to be involved in various biological processes. METTL3 can enhance the BAT-mediated adaptive thermogenesis and suppress obesity and systemic insulin resistance via targeting the 3′ UTR of the PRDM16, PPARG, and UCP1 transcript to install the m^6^A modification (Wang Y. et al., 2020). The ablation of METTL3 in germ cells severely inhibited spermatogonial differentiation and blocked the initiation of meiosis (Xu et al., 2017). Besides, METTL3 was also shown to be upregulated in various solid tumors and associated with poor prognosis. In oral squamous cell carcinoma (OSCC), METTL3 can facilitate tumor growth and metastasis through making an increment in m^6^A modification and expression of c-Myc transcript (Zhao W. et al., 2020). In colorectal cancer (CRC), METTL3 stabilizes HK2 and GLUT1 expression via a m^6^A -IGF2BP2/3-dependent mechanism (Shen et al., 2020). Additionally, METTL3 might affect tumor metastasis through promoting the maturation of pri-miR-1246 (Peng et al., 2019). METTL3 enhances the splicing of precursor miR-143-3p and facilitates its biogenesis, thereby promoting the brain metastasis of lung cancer (LC) (Wang H. et al., 2019). Moreover, METTL3 induces non-small cell lung cancer (NSCLC) drug resistance and metastasis by promoting Yes-associated protein (YAP) mRNA translation via a m^6^A -YTHDF1/3/eIF3b-dependent mechanism (Jin D. et al., 2019). In gastric cancer (GC), overexpression of METTL3 can promote the stability of ZMYM1, thereby enhancing epithelial mesenchymal transformation (EMT) process and tumor metastasis (Yue et al., 2019). In addition, upregulated METTL3 facilitates GC growth and liver metastasis through installing m^6^A modifications of HDGF transcript (Wang Q. et al., 2020).

## METTL14

Studies have demonstrated that METTL14 is associated with a lower risk for development of neoplasms. In CRC, METTL14 acts as a tumor-suppressor to inhibit cell growth and metastasis *in vitro* and *in vivo*. Mechanistical study demonstrated that downregulated METTL14 substantially abolishes m^6^A modifications of XIST and augments XIST expression (Yang X. et al., 2020). In addition, METTL14 can inhibit CRC cell proliferation, migration and invasion via the miR-375-YAP1/SP1 signal axis (Chen X. et al., 2020). Although both of METTL3 and METTL14 could act as m^6^A “writer”, METTL3 might promote the progression of CRC, while METTL14 functions as a tumor suppressor in CRC. METTL14 can also assume an oncogenic role in triple-negative breast cancer (TNBC) (Shi et al., 2020), pancreatic cancer (Kong et al., 2020) and leukemia (Weng et al., 2018). Moreover, METTL14 is significantly upregulated in Epstein–Barr virus (EBV) latently infected cells. METTL14 can lead to oncogenesis via increasing m^6^A modifications of the indispensable EBV latent antigen EBNA3C and thus facilitating its stability and expression. Interestingly, EBNA3C can also enhance stability and expression of METTL14 (Lang et al., 2019).

## METTL16

METTL16 has been recently shown to have distinct target RNAs for m^6^A modification. Studies have revealed that METTL16 can bind a subset of mRNAs and methylate U6 small nuclear RNA (U6 snRNA) and long non-coding RNA (lncRNA) (Brown et al., 2016; Fitzsimmons and Batista, 2019). Moreover, the UACAGAGAA sequence is essential for METTL16-mediated-methylation and the Nterminal module of METTL16 is required for RNA binding (Doxtader et al., 2018; Mendel et al., 2018). METTL16 is involved in catalyzing m^6^A in A43 of the U6 small nuclear RNA (Warda et al., 2017). Under loss-of-SAM conditions, METTL16 can induce the splicing of a retained intron, thereby enhancing level of MAT2A and expression of SAM, while down-regulation of METTL16 and YTHDC1 can abolish SAM-responsive regulation of MAT2A (Pendleton et al., 2017; Shima et al., 2017). While the specific role of METTL16 in solid tumors remain to be further explored.

### Demethylases (“Erasers”)

The reversible and dynamic m^6^A modification can be mediated by obesity-associated protein (FTO) and alkB homolog 5 (ALKBH5) (m^6^A “erasers”) (Jia et al., 2011; Zheng et al., 2013). Both FTO and ALKBH5 are members of the ALKB family of dioxygenases. As the first reported demethylase, FTO can also mediate demethylation of both N6-hydroxymethyladenosine (hm^6^A) and N6-formyladenosine (f6A) in mRNA (Basak et al., 2019). ALKBH5 plays an essential role in mRNA export and RNA metabolism (Tang et al., 2018).

## FTO

As an m^6^A eraser, FTO is associated with the initiation and development of various cancers including hepatocellular carcinoma (HCC), melanoma, breast cancer and glioma. In HCC, SIRT1 destabilizes FTO and thus steering the m^6^A of downstream elements and consecutive mRNA expression in tumorigenesis (Liu X. et al., 2020). In melanoma, FTO can impair IFNγ-induced killing via augmenting CXCR4, PD-1 and SOX10 expression via repressing YTHDF2-mediated degradation and suppress response to anti-PD-1 blockade immunotherapy (Yang S. et al., 2019). In breast cancer, FTO enhances breast cancer cell growth, colony formation and metastasis. Mechanistical study demonstrated that FTO can mediate m^6^A demethylation of BNIP3 transcript and induce its degradation via an YTHDF2 independent mechanism (Niu et al., 2019). The ethyl ester form of meclofenamic acid (MA2) inhibits FTO and enhances the effect of the chemotherapy drug temozolomide (TMZ) on suppressing proliferation of glioma cells (Xiao et al., 2020).

## ALKBH5

ALKBH5 has been regarded as a tumor suppressor in many cancers. In NSCLC, ALKBH5 suppresses cell growth and metastasis both *in vitro* and *in vivo* via repressing miR-107/LATS2-mediated YAP activity and YTHDFs-mediated YAP expression (Jin D. et al., 2020). In pancreatic cancer, downregulated ALKBH5 predicts poor prognosis and knockdown of ALKBH5 markedly facilitates tumor growth and metastasis (Tang et al., 2020). In HCC, ALKBH5 is characterized as a tumor suppressor and could attenuate the expression of LYPD1 via an mA-dependent manner in HCC cells (Chen Y. et al., 2020). In addition, ALKBH5 can augment steady-state CYR61 mRNA expression via an m^6^A -dependent mechanism, thereby repressing trophoblast invasion (Li X. C. et al., 2019).

### m^6^A Binding Proteins (“Readers”)

**FIGURE 2:**
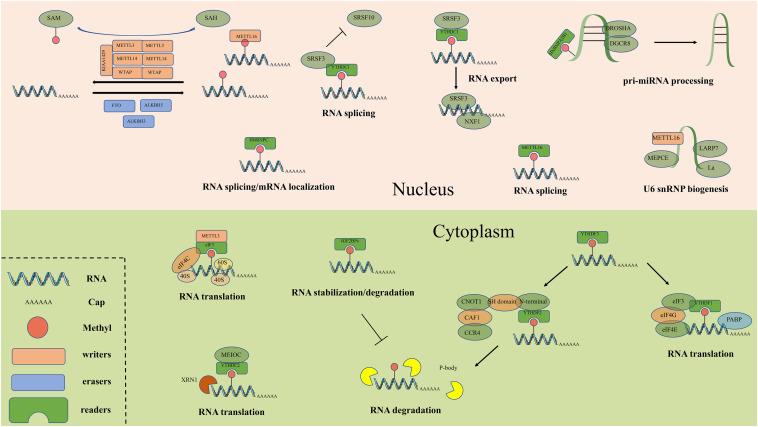
The detailed molecular mechanism of m^6^A enzymes. The “writers”, “erasers” and “readers” relay on a variety of related factors install, remove and recognize m^6^A mutation and participate in RNA metabolic processes, including translation, splicing, export, degradation and so on.

M^6^A readers can recognize and bind to m^6^A sites and regulate target RNA translation, splicing, nuclear export and decay (Figure 2). In YTH (YT521-B homology) domain family, the evolutionarily conserved YTH domain acts as the module for directly binding to m^6^A. YTHDF1–3 and YTHDC1–2 are the main five YTH domain proteins. YTHDF1 can bind to m^6^A sites around the stop codon and thus facilitating mRNA translation efficiency (Liu X. et al., 2020). YTHDF2 can accelerate degradation and deadenylation of the transcripts by bringing m^6^A-modified translatable mRNAs to mRNA decay sites and recruiting CCR4-NOT deadenylase complex (Zhou J. et al., 2015). YTHDF3 can, respectively, promote RNA translation through associating with YTHDF1 and enhance RNA degradation by interacting with YTHDF2 (Li A. et al., 2017; Shi et al., 2017). In contrast to the prevailing model, where each DF paralog binds to distinct subsets of mRNAs, Zaccara and Jaffrey show that the DF paralogs bind proportionately to each m^6^A site throughout the transcriptome (Zaccara and Jaffrey, 2020). YTHDC1 recruits the RNA splicing and control the nuclear export (Roundtree et al., 2017b). YTHDC2 interacts with RNA helicase and increases the translation efficiency of target RNA (Mao et al., 2019). The insulin-like growth factor 2 mRNA binding protein (IGF2BP) family proteins, including IGF2BP1-3, can recognize m^6^A containing transcripts. IGF2BPs exert their functions via recruiting RNA stabilizers (Huang H. et al., 2018). Eukaryotic initiation factor 3 (EIF3) can facilitate cap-independent translation (Meyer et al., 2015). Heterogeneous nuclear ribo nucleo protein (HNRNP) family proteins include hnRNPC, hnRNPG and hnRNPA2B1. HnRNPC and hnRNPG can influence alternative splicing and mRNA localization (Guichard et al., 2012) while hnRNPA2B1 can bind to m^6^A -containing primary microRNAs and enhance microRNA maturation (Alarcon et al., 2015).

## YTHDF1

More recently, YTHDF1 has been proved to be upregulated in various tumors, associated with more advanced stages and poorer survival. In ovarian cancer, YTHDF1 promotes tumor growth and metastasis. Mechanistically, YTHDF1 binds to the m^6^A modification site of EIF3C 3′-UTR to increase the translation of EIF3C mRNA (Liu X. et al., 2020). YTHDF1 could promote the translation of frizzled7 (FZD7) in an m^6^A-dependent manner, leading to hyper-activation of the Wnt/β-catenin pathway and promotion of gastric carcinogenesis (Pi et al., 2020). Besides, YTHDF1 binds the m^6^A modification site of Robo3.1 3′-UTR and promotes its translation in an m^6^A-independent mechanism. While down-expression of YTHDF1 in spinal commissural neurons contributes to pre-crossing axon guidance defects (Zhuang M. et al., 2019).

## YTHDF2

Evidence has shown that YTHDF2 can act as an oncogene or tumor suppressor in different tumor models. In HCC, YTHDF2 decreased expression level is associated with poor prognosis and classification. YTHDF2 may participate in the occurrence and progression of HCC by processing the decay of m^6^A-containing serpin family E member 2 (SERPINE2) and interleukin 11 (IL11) mRNAs (Hou et al., 2019). Besides, YTHDF2 can suppress tumor growth through modulating the m^6^A methylation of EGFR mRNA by the m^6^A/mRNA degradation pathway. However, YTHDF2 promotes the cancer stem cell liver phenotype and cancer metastasis by binding m^6^A-modified OCT4 mRNA (Zhang et al., 2020). YTHDF2 can also interact with miRNA, miR-145 targets YTHDF2 and results in its degradation (Yang Z. et al., 2017). Moreover, YTHDF2 is also involved in the initation of other biological process. In spermatogenesis, YTHDF2 regulates cell proliferation and adhesion via modulating the m^6^A methylation of MMPs and simultaneously decreasing the overall translational output (Huang T. et al., 2020). Knockdown of YTHDF2 promotes the expression of MAP2K4 and MAP4K4 and activates MAPK and NF-κB signaling pathways, which facilitate the expression of proinflammatory cytokines and exacerbate the inflammatory response in LPS-stimulated RAW 264.7 cells (Yu et al., 2019).

## YTHDF3

YTHDF3 has been reported to play a fine-tuning role in the RNA accessibility of YTHDF1 and YTHDF2 and biological process. In CRC, lncRNA GAS5 leads to ubiquitin-mediated degradation of YAP via interacting with WW domain of YAP, thus repressing tumor progression. While YTHDF3 might recognize m^6^A-modified GAS5 and induce decay of it (Ni et al., 2019). YTHDF3 can serve as a negative regulator to enhance the translation of FOXO3 mRNA, thereby maintaining host antiviral immune function and preventing inflammatory response (Zhang Y. et al., 2019).

## YTHDC1

YTHDC1 and YTHDC2 have conserved m^6^A binding domain and preferentially bind to m^6^A-modified RNA in RRm6ACH consensus sequence (Roundtree et al., 2017a). YTHDC1 is involved in processing of pre-mRNA transcripts of F6, SRSF3, and SRSF7 in the oocyte nucleus, and it may play a crucial role in fetal development (Kasowitz et al., 2018). MAT2A mRNA can be methylated by METTL16 and YTHDC1 can bind to the m^6^A modification site of MAT2A 3′-UTR. Downregulation of METTL16 and YTHDC1 might effectively abolish SAM-responsive regulation of MAT2A (Shima et al., 2017). The m^6^A modification site of long non-coding RNA X-inactive specific transcript (XIST) can be preferentially read by YTHDC1 and it’s required for XIST function (Patil et al., 2016). Recent study shows that the ability of the YTH domain of YTHDC1 binding to ssDNA is stronger than in an RNA context. However, the YTH domains of YTHDF2 and YTHDF1 exhibit the opposite effect (Woodcock et al., 2020).

## YTHDC2

YTHDC2 could bind mitotic transcripts, specific piRNA precursors and interact with RNA granule components, licensing the proper progression of germ cells through meiosis (Bailey et al., 2017). YTHDC2 results in colon cancer metastasis through augmenting translation of HIF-1α, it may be a potential diagnostic marker and therapy target in colon cancer (Tanabe et al., 2016). YTHDC2 binds to the mRNA of lipogenic genes and participates in the regulation of hepatic lipogenesis and TG homeostasis (Zhou B. et al., 2020).

## IGF2BPs

IGFBPs could use common RNA binding domains to recognize m^6^A containing transcripts and play a significant role in many diseases. In breast cancer, FGF13-AS1 can reduce the half-life of c-Myc (Myc) mRNA by binding IGF2BPs, thus suppressing cell proliferation, migration and invasion (Ma et al., 2019). In ovarian cancer, IGF2BP1 enhances cell proliferative and invasive ability by antagonizing miRNA-impaired gene expression, the elevate expression of IGF2BP1 is correlated to poor prognosis (Muller et al., 2018). IGF2BP1 could function as an adaptor protein to recruit the CCR4-NOT complex, so as to initiate the degradation of the lncRNA highly up-regulated in liver cancer (HULC) (Hammerle et al., 2013). In pancreatic cancer, IGF2BP2 could promote cell growth through activating the PI3K/Akt signaling pathway and be negatively regulated by miR-141 (Xu et al., 2019). In addition, IGF2BP2 enhances cancer stemness-like properties and promotes tumorigenesis by acting as a reader for m^6^A modified DANCR (Hu et al., 2020). In gastric cancer, miR-34a directly targets IGF2BP3, overexpression of IGF2BP3 promotes cell proliferation and invasion (Zhou Y. et al., 2017). IGF2BP3 could interact with RNA-binding protein Lin28b and thereby promotes stability and expression of target mRNAs such as B-cell regulators Pax5 and Arid3a, so as to participate in the fetal–adult hematopoietic switch (Wang S. et al., 2019).

## EIF3

EIF3 is crucial for specialized translation initiation via interacting with the 5′; cap region, resulting in assemblage of translation initiation complexes on eIF3-specialized mRNA (Lee et al., 2016). Study has proved that YTHDF1 might promote the translation of EIF3 via recognizing the m^6^A-modified sites of EIF3 mRNA and simultaneously augments the overall translational output, thus facilitating tumorigenesis and metastasis in ovarian cancer (Liu X. et al., 2020). In renal cell carcinoma (RCC), knockdown of EIF3 dramatically decreases cell viability with sunitinib treatment. Mechanistically, EIF3 could interact with GRP78 and enhance protein stability by blocking the ubiquitin-mediated degradation of GRP78 (Huang H. et al., 2019). In gallbladder cancer (GBC), EIF3 can stabilize GRK2 protein through blocking ubiquitin-mediated degradation, wherefore activating PI3K/Akt signal pathway and enhancing tumor growth and metastasis (Zhang et al., 2017). All above studies demonstrate that EIF3 is a vital role in the progerssion of various cancers.

## Roles of RNA m^6^A in Urological Tumors

**FIGURE 3:**
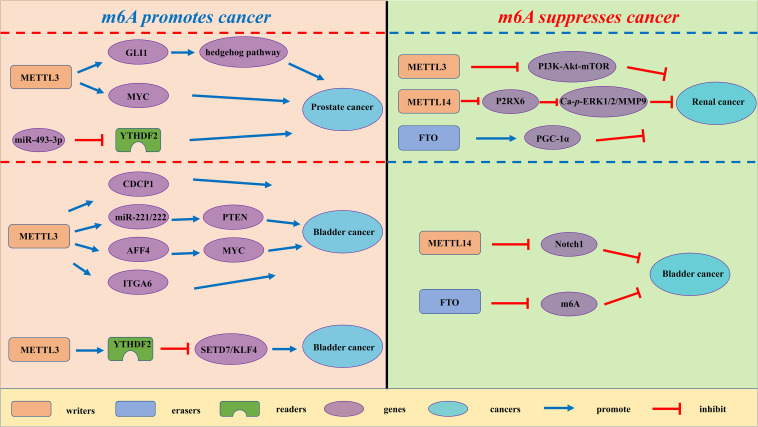
The potential roles of m^6^A in urological tumors progression. The potential role of m^6^A in urological tumors progression is reflected in the regulation of tumor-associated gene expression. M^6^A modification promotes urological tumors progression by enhancing oncogene expression and inhibiting tumor suppressor gene expression. M^6^A modification hinders cancer progression by inhibiting oncogene expression and enhancing tumor suppressor gene expression.

Accumulating evidence indicates that RNA m^6^A modification is related to the tumorigenesis, development and progression of urological tumors. Therefore, we summarize these latest advances of m^6^A modification in urological tumors (Table 2, Figure 3).

**TABLE 2:** The roles of RNA m^6^A in urological tumors.

Cancer	m^6^A Regulators	Role in cancer	Biological function	Mechanism	References
Renal cancer	METTL3	Suppressor	Suppresses RCC proliferation, migration	Regulates EMT and PI3K-Akt-mTOR pathways	Rini et al., 2009
		gene	and invasion		
–	METTL14	Suppressor	Suppresses RCC migration and invasion	Down-regulates P2RX6 protein translation	Li X. et al., 2017
–	–	gene	–	–	–
–	–	–	–	–	–
	FTO	Suppressor	Spppress RCC growth	Promotes PGC-1α expression by reducing m^6^A levels	Gong et al., 2019
		gene			
Prostate cancer	METTL3	Oncogene	Promotes PCa growth and metastasis	Regulates hedgehog pathway	Siegel et al., 2020
–	–	–	–	–	–
	METTL3	Oncogene	Promotes PCa proliferation, migration	Promotes MYC expression by increasing m6A levels	Cai et al., 2019
			and invasion		
–	YTHDF2	Oncogene	Promotes PCa proliferation and migration	/	Yuan et al., 2020
–	–	–	–	–	–
Bladder cancer	METTL3	Oncogene	Promotes BC growth	Promotes CDCP1 mRNA modification and translation	Bray et al., 2018
–	METTL3	Oncogene	Promotes BC proliferation	Interactes with the microprocessor protein DGCR8 and	Yang F. et al., 2019
–	–	–	–	positively modulates the pri-miR221/222 process	–
–	–	–	–	–	–
	METTL3	Oncogene	Promotes BC growth and metastasis	Regulates AFF4/NF-κB/MYC signaling network	Han et al., 2019
–	METTL3	Oncogene	Promotes BC growth and metastasis	promote the translation of ITGA6 mRNA	Cheng et al., 2019
–	–	–	–	–	–
	METTL3/YTHDF2	Oncogene	Promotes BC growth and metastasis	METTL3/YTHDF2 may mediate the mRNA decay	Jin H. et al., 2019
				of tumor suppressors SETD7 and KLF4	
–	METTL14	Suppressor	Promotes the proliferation, self-renewal, metastasis	METTL14 knockdown may enhance the RNA	Xie et al., 2020
–	–	gene	and tumor initiating capacity of bladder TICs	stability of Notch1 mRNA	–
–	–	–	–	–	–
	FTO	Suppressor	Inhibits BC proliferation and migration	/	Gu et al., 2019
		Gene			

### Renal Cell Carcinoma

Renal cell carcinoma (RCC) is derived from renal epithelium and is one of the most common cancers worldwide, making up nearly 2% to 3% of all adult malignancies (Rini et al., 2009). Li and collaborators demonstrated that METTL3 was a potential prognostic marker of RCC, and the expression levels of METTL3 are interrelated to tumor size and histological grade. Inhibition of METTL3 could obviously promote cell proliferation, migration and invasion, and make cell cycle arrest (Li X. et al., 2017). In addition, METTL3 knockdown might activate oncogenic PI3K/Akt/mTOR signaling pathway. Hence, METTL3 might function as a tumor suppressor in the tumorigenesis of RCC. Gong and co-workers found that the expression level of METTL14 is decreased in RCC (Gong et al., 2019). Additionally, the mRNA level of METTL14 is associated with RCC patients’ overall survival. Knockdown of METTL14 promotes the mRNA and protein expression levels of P2RX6, while P2RX6 could further regulate the Ca^2+^-mediated p-ERK1/2/MMP9 signal pathway promote cell migration and invasion. Zhuang C. et al. (2019) found that PGC-1α underwent m^6^A methylation in RCC. As an m^6^A demethylase, FTO could recognize the m^6^A sites of PGC-1α and reduce its methylation level, therefore leading to the increases in mitochondria biogenesis and oxidative phosphorylation and the decreases in tumor growth of RCC (Zhuang C. et al., 2019).

### Prostate Cancer

Prostate cancer (PCa) has been regarded as the most common cancer among men and the second cancer-related deaths in the men in 2019 (Siegel et al., 2020). Despite recent advances in many therapies, the 5 years’ survival rate for prostate cancer patients remains low. Cai et al. found that METTL3 is overexpressed in PCa tissues and cell lines (Cai et al., 2019). Elevated expression of METTL3 could promote cell proliferation, survival, colony formation, and invasion. Moreover, knockdown of METTL3 could decrease the m^6^A modification and expression of GLI1, thereby regulating hedgehog pathway. Yuan et al. (2020) demonstrated that the mRNA expression level of METTL3 was increased in prostate cancer tissues. Additionally, the expression level of METTL3 is associated with the deterioration of PCa patients’ condition. Mechanistically, METTL3 could enhance MYC (c-myc) expression via elevating m^6^A levels of MYC mRNA transcript, so as to facilitate the proliferative, migrative and invasive ability of cancer cells. Li found that YTHDF2, an m^6^A reader, was upregulated in prostate cancer tissues and cell lines (Li J. et al., 2018). Knockdown of YTHDF2 led to decreased levels of m^6^A and impaired proliferation and migration of PCa cells. Therefore, YTHDF2 played a vital role in the initition and progression of PCa.

### Bladder Cancer

Bladder cancer (BCa) is the most common urogenital and the 10th most common cancer worldwide, with an estimated 549 000 new cases and 200 000 deaths in 2018 (Bray et al., 2018). Despite the improvement of clinical diagnosis and therapies, BCa is regarded as a major cause of cancer-interrelated morbidity and mortality. In the study of Yang F. et al. (2019) the expression levels of METTL3 were elevated in BCa patient samples. The increase in METTL3 expression was proven to be correlated with BCa growth and progression *in vitro* and *in vivo*. Moreover, METTL3 could positively regulate CDCP1 process based on an m^6^A -dependent mode, bringing about elevated expression of CDCP1. Han et al. (2019) demonstrated that the expression level of METTL3 in BCa was significantly up-regulated and associated with poor prognosis of BCa patients. They found that METTL3 might interact with the microprocessor protein DGCR8 and positively modulate the pri-miR221/222 process through an m^6^A -dependent mechanism. Cheng and coworkers elucidated that METTL3 was obviously up-regulated in BCa tissues and significantly promoted growth and metastasis of BCa (Cheng et al., 2019). Mechanistically, METTL3 might promote BCa progression via AFF4/NF-κB/MYC signaling pathway. Jin and coworkers demonstrated that METTL3 and ALKBH5 can alter cell adhesion via regulating ITGA6 expression in BCa (Jin H. et al., 2019). Increased m^6^A methylation enhanced the translation of ITGA6 mRNA by binding of YTHDF1 and YTHDF3 and promoted malignant phenotypes in BCa. Xie and coworkers found that knockout of METTL3 impaired tumor growth and metastasis, METTL3/YTHDF2 m^6^A axis could directly degrad the mRNA expression of the tumor suppressors SETD7 and KLF4, leading to the development and progression of BCa (Xie et al., 2020). Gu et al. (2019) found a decrease of N6-methyladenosine in BCa and bladder tumor initiating cells (TICs). In addition, METTL14 is down-regulated in BCa and bladder TICs and it could promote the proliferation, metastasis, self-renewal and enhance tumor initiating capacity of bladder TICs. Mechanistically, METTL14 might regulate Notch1 expression in an m^6^A-dependent manner. Wen demonstrated that knockdown of FTO could accelerate the progression of BCa (Wen et al., 2020), while the potential mechanism remains unknown.

### Testicular Germ Cell Tumors

Testicular germ cell tumors (TGCTs) are the most common solid neoplasm among men aged between 14 and 44 years (Cheng et al., 2018). Despite the advanced prognosis of localized TGCTs, approximately 20–30% of patients may experience disease recurrence during surveillance (Mortensen et al., 2016). Lobo and coworkers demonstrated that abundance of m^6^A and expression of VIRMA/YTHDF3 were different among TGCTs subtypes, with higher levels in seminomas (SEs), suggesting a contribution to SE phenotype maintenance (Lobo et al., 2019). However, the potential biological roles of VIRMA/YTHDF3 remain to be further explored.

### Wilms Tumor

Wilms tumor (WT) is the most prevalent childhood kidney tumor characterized by the disorganized and dysregulated development of a kidney (Davidoff, 2009; Servaes et al., 2019). Hua et al. (2020) found an obvious relationship between ALKBH5 rs1378602 AG/AA genotypes and decreased Wilms tumor risk in children in clinical stage I diseases. However, the observed association should be further validated in another well-designed analysis with other larger ethnicities.

## Potential Application of RNA M^6^A in Urological Tumors Rna

### RNA m^6^A as Biomarker in Urological Tumors

Mounting evidence has indicated that m^6^A regulators have the potential to be superior diagnostic and prognostic biomarkers for urological tumors patients. Strick et al. conducted qRT-PCR to detect the gene expressions of ALKBH5 and FTO were studied in 166 ccRCC and 106 normal renal tissues. They found that the expression level of ALKBH5 and FTO were obviously decreased in ccRCC tissues (Strick et al., 2020). Declined mRNA levels of ALKBH5 and FTO were related to a shortened overall and cancer-specific survival following nephrectomy. Therefore, ALKBH5 and FTO could be used as prognostic biomarkers for RCC. Zhao Y. et al. (2020) demonstrated that METTL14 mRNA expression negatively correlated with the RCC stages and positively correlated with RCC patients’ overall survival, it might be a potential biomarker of RCC. Yuan et al. performed the qRT-PCR to detect the mRNA expression level of METTL3 in 84 clinical human PCa specimens and 32 corresponding adjacent normal specimens. The results showed that a significant positive association between METTL3 expression was observed with tumor stage and metastasis. Moreover, the expression level of METTL3 had remarkable prognostic value for overall survival and disease-free survival (Yuan et al., 2020); hence, METTL3 might play a vital role in PCa progression and metastasis. Chen et al. concluded that m^6^A regulators were related to malignant clinicopathological features of BCa and a risk signature with FTO, WTAP and YTHDC3 might play vital roles in diagnosis and prognosis of BCa patients (Chen M. et al., 2019). In TGCTs, VIRMA and YTHDF3 might be prognostic factors (Lobo et al., 2019).

### RNA m^6^A as Therapeutic Targets in Urological Tumors

The critical roles of m^6^A in urological tumors suggest that it has the potential to be involved in tumor therapy. A number of studies have indicated that m^6^A modification is significant in therapies of urological tumors, especially in targeted treatment. Zhuang et al. found that the Von Hippel-Lindau (VHL) -deficient cells expressing FTO might restore mitochondrial activity, induce oxidative stress and ROS production and suppressed tumor growth, via promoting PGC-1α expression by decreasing m^6^A levels in its mRNA transcripts (Zhuang C. et al., 2019). Therefore, the m^6^A methylation and m^6^A-related regulators, and uncovers an essential FTO-PGC-1α axis might play a vital role in the treatment of RCC. Gong and coworkers found that ATP could enhance cell migration and invasion via rugulating P2RX6 expression in RCC (Gong et al., 2019). Mechanistically, ATP-P2RX6 could modulate the Ca^2+^-mediated p-ERK1/2/MMP9 signal pathway, while METTL14 might down-regulate P2RX6 protein translation in an m^6^A-dependent manner. Herein, the further exploration of regulation of METTL14 expression might contribute to develop a new approach to repress RCC progression. Li et al. suggested that METTL3 expression is higher in PCa than in normal prostate tissues, especially in PCa with bone metastasis (Li E. et al., 2020). METTL3 regulates the expression of Integrin β1 (ITGB1) through m^6^A-HuR-dependent mechanism, which affects the binding of ITGB1 to Collagen I and tumor cell motility, so as to promote the bone metastasis of PCa. Therefore, METTL3 might act as a therapeutic target for PCa bone metastasis. Wen et al. found that knockdown of FTO could enhance cell proliferation and migration and protect BCa cells from cisplatin-induced cytotoxicity (Wen et al., 2020). Hence, targeting the m^6^A modification of FTO may be beneficial to the treatment of BCa.

## Discussion

Recently, RNA epigenetics is emerging as a hot topic. Among them, m^6^A modification has become a new layer of post-transcriptional regulation of gene expression. The implications of m^6^A modifications in human carcinogenesis have been verified in many kinds of cancers, including urological tumors. In this review, we summarized the potential biological effects of m^6^A-related regulators, and particularly focused on the impacts of m^6^A modification on different tumors in the urinary system. M^6^A can be installed by the methyltransferase, while these modifications may be removed by m^6^A eraser demethylases. Furthermore, m^6^A readers could specifically recognize the m^6^A methylation sites and thus regulating mRNA splicing, translation, degradation, nuclear export, and other cellular processes. Besides, m^6^A methylation and its related regulatory factors are reported to be involved in the processing and the biological function of non-coding coding RNAs (Coker et al., 2019; Huang H. et al., 2020).

However, m^6^A methylation seems to serve as a double-edged sword due to the specific mechanism for m^6^A in cancers remains unknown. Some genes may lead to cancer progression after m^6^A methylation, while removal of m^6^A modification can result in the progression of other tumors. For example, in HCC, sumo1 modification of METTL3 can promote tumor progression via regulating snail mRNA homeostasis (Xu et al., 2020), while in glioblastoma, LncRNA SOX2OT can facilitate temozolomide resistance through promoting SOX2 expression via ALKBH5-mediated epigenetic regulation (Liu X. et al., 2020). In addition, the same m^6^A-associated regulator may play crucial roles in the same type of cancer via targeting different downstream genes. For instance, in CRC, METTL3 can promote tumor progression through enhancing the expression of either MYC (Xiang et al., 2020) or CCNE1 (Zhu W. et al., 2020). Additionally, researches have reported conflicting findings in the same type of cancer; for instance, in CRC, METTL3 and METTL14 play totally opposite roles in tumor initition and progression (Li T. et al., 2019; Yang X. et al., 2020). Overall, all above studies show that m^6^A methylation and its related regulatory networks are complex and need to be further explored. Moreover, Han et al. found that METTL3 can enhance tumor growth of BCa through accelerating pri-miR221/222 maturation based on m^6^A-dependent mode (Han et al., 2019), while Gu et al. (2019) reported that METTL14 can inhibit bladder tumorigenesis through N6-methyladenosine of Notch1. The above discrepancy may result from several factors such as case sample size and different related regulatory genes. Furthermore, studies have identified the therapeutic potential of m^6^A modification. METTL3 might induce NSCLC drug resistance and metastasis via modulating the MALAT1-miR-1914-3p-YAP axis (Jin D. et al., 2019). In glioma, METTL3 can promote glioma radioresistance and stem-like cell maintenance (Visvanathan et al., 2018). In melanoma, FTO can act as an m^6^A demethylase to promote melanoma tumorigenesis and anti-PD-1 resistance (Yang S. et al., 2019). R-2HG can inhibit FTO activity and thus elevating m^6^A mRNA modification in R-2HG-sensitive leukemia cells, thereby generating anti-leukemia effects (Su et al., 2018). In cervical squamous cell carcinoma (CSCC), FTO can regulate the chemo-radiotherapy resistance by targeting β-catenin through mRNA demethylation (Zhou S. et al., 2018).

The advanced development of m^6^A modification study marks a novel insight in the dignosis and therapy of various diseases. Nevertheless, we believe that future prospects on m^6^A modification need to be further explored. Firstly, several databases (such as GEPIA, TCGA et al.) were used in many studies to explore the prognostic significance of m^6^A regulators expression in OS and DFS of urological tumors patients. Hence, expansion of the sample size and screening factors are essential for early diagnosis and prognosis; while the specificity and sensitivity of m^6^A-related regulators also need to be discussed. Secondly, more and more clinical pratice are urgent for confirming the therapeutic potential of m^6^A regulatory factors and related pathways. Thirdly, it’s significant to construct a complex and specific regulatory network model of m^6^A and its associated modifiers in a single cancer. Fourthly, exploring other components of m^6^A methylation and demethylation and effectors is necessary.

## Conclusion

Urological tumors are major public health concern with growing prevalence. Studies have showed that m^6^A methylation plays a significant role in prevention, treatment and management of various urological tumors; however, more endeavors and more multi-center and large-scale research are urgent for exploring the relationship between m^6^A modification and urological tumors.

## Author Contributions

YL and YG collected the related manuscript and finished the manuscript and figures. RJ gave constructive guidance and made final approval. LX, ZX, and QD participated in the design of this review. All authors read and approved the final manuscript.

## Conflict of Interest

The authors declare that the research was conducted in the absence of any commercial or financial relationships that could be construed as a potential conflict of interest.
